# The Case for TAAR1 as a Modulator of Central Nervous System Function

**DOI:** 10.3389/fphar.2017.00987

**Published:** 2018-01-10

**Authors:** Grazia Rutigliano, Alice Accorroni, Riccardo Zucchi

**Affiliations:** ^1^Istituto di Scienze della Vita, Scuola Superiore Sant’Anna, Pisa, Italy; ^2^Institute of Clinical Physiology, National Research Council, Pisa, Italy; ^3^Department of Pathology, University of Pisa, Pisa, Italy

**Keywords:** TAAR1, T1AM, dopamine, neurotransmitter hormone, trace amines

## Abstract

TAAR1 is widely expressed across the mammalian brain, particularly in limbic and monoaminergic areas, allegedly involved in mood, attention, memory, fear, and addiction. However, the subcellular distribution of TAAR1 is still unclear, since TAAR1 signal is largely intracellular. *In vitro*, TAAR1 is activated with nanomolar to micromolar affinity by some endogenous amines, particularly *p*-tyramine, beta-phenylethylamine, and 3-iodothyronamine (T1AM), the latter representing a novel branch of thyroid hormone signaling. In addition, TAAR1 responds to a number of psychoactive drugs, i.e., amphetamines, ergoline derivatives, bromocriptine and lisuride. Trace amines have been identified as neurotransmitters in invertebrates, and they are considered as potential neuromodulators. In particular, beta-phenylethylamine and *p*-tyramine have been reported to modify the release and/or the response to dopamine, norepinephrine, acetylcholine and GABA, while evidence of cross-talk between TAAR1 and other aminergic receptors has been provided. Systemic or intracerebroventricular injection of exogenous T1AM produced prolearning and antiamnestic effects, reduced pain threshold, decreased non-REM sleep, and modulated the firing rate of adrenergic neurons in locus coeruleus. However each of these substances may have additional molecular targets, and it is unclear whether their endogenous levels are sufficient to produce significant TAAR1 activation *in vivo*. TAAR1 knock out mice show a worse performance in anxiety and working memory tests, and they are more prone to develop ethanol addiction. They also show increased locomotor response to amphetamine, and decreased stereotypical responses induced by apomorphine. Notably, human genes for TAARs cluster on chromosome 6 at q23, within a region whose mutations have been reported to confer susceptibility to schizophrenia and bipolar disorder. For human TAAR1, around 200 non-synonymous and 400 synonymous single nucleotide polymorphisms have been identified, but their functional consequences have not been extensively investigated yet. In conclusion, the bulk of evidence points to a significant physiological role of TAAR1 in the modulation of central nervous system function and a potential pharmacological role of TAAR1 agonists in neurology and/or psychiatry. However, the specific effects of TAAR1 stimulation are still controversial, and many crucial issues require further investigation.

## Introduction

Trace amine-associated receptor 1 (TAAR1) was identified in 2001 through a degenerate PCR approach: by using primers based on the sequences of dopamine or serotonin receptors, a novel G protein-coupled receptor (GPCR) was discovered, which turned out to respond to some trace amines, rather than to classical biogenic amines, and was at that time named trace amine receptor 1 (TA1 or TAR1) (Borowsky et al., 2001; Bunzow et al., 2001). The term “trace amine” needs some clarification. It was introduced to designate endogenous amines whose tissue concentrations are physiologically < 100 ng/g tissue (Boulton, 1974), and it was initially applied to *p*-tyramine (TYR), 2-phenylethylamine (PEA), and tryptamine. These amines are produced by the decarboxylation of aromatic amino acids (respectively, tyrosine, phenylalanine, and tryptophan) which is assumed to be catalyzed by the enzyme aromatic amino acid decarboxylase (AADC) (Berry, 2004). Although other amines have been detected in tissues at very low concentrations, in its current use the term is still restricted to the three original compounds, and to some of their derivatives, namely 2-hydroxy-*p*-tyramine (octopamine), *N*-methyl-2-hydroxy-*p*-tyramine (synephrine), and 3-methoxy-*p*-tyramine.

Homology analysis led to the conclusion that TAAR1 is the prototype of a novel class of aminergic receptors. However, it became evident that some members of this family have different pharmacological profiles. Therefore, it was suggested to rename them as “trace amine-associated receptors,” and the acronym TAAR was introduced (Lindemann et al., 2005). This term has been accepted by the Human Genome Organization (HUGO) Gene Nomenclature Committee and will be used in this review, although the International Union of Pharmacology (IUPHAR) still recommends the original denomination of “trace amine receptors” (Maguire et al., 2009).

Up to 28 distinct TAAR subfamilies have been described so far. The TAAR1-9 subfamilies are expressed in most vertebrates, while the TAAR10-28 subfamilies have only be detected in teleosts (Gloriam et al., 2005; Hashiguchi and Nishida, 2007; Hussain et al., 2009). Notably, TYR and octopamine are thought to be the chief neuromodulators in insects (Roeder, 1999; Grohmann et al., 2003), but there appears to be no phylogenetic relation between vertebrate TAARs and invertebrate TYR and octopamine receptors.

The large number of TAARs, and their wide distribution in all vertebrate phyla, are consistent with a major biological role, but their specific function has not been determined yet, and the endogenous agonists responsible for their physiological activation have not been definitely identified. Some TAARs appear to be olfactory receptors, at least in rodents (Liberles and Buck, 2006), and a distinction between “olfactory” and “non-olfactory” TAARs has been proposed. It is quite possible that, during vertebrate evolution, molecules able to bind the products of aromatic amino acid decarboxylation have progressively developed the capacity to interact with different ligands, acquiring novel functional roles.

The biochemical and biological features of TAARs have been discussed in several excellent reviews (Lindemann and Hoener, 2005; Lindemann et al., 2005; Lewin, 2006; Zucchi et al., 2006; Grandy, 2007; Liberles, 2015; Berry et al., 2017). In the present paper, we will focus on a single issue, namely the elusive relationship between TAAR1 and central nervous system (CNS) function. As a matter of fact, TAAR1 has been initially identified in the CNS, and several lines of evidence have implied it in the control of neuronal interaction. However, many crucial questions are still open, and the alleged role of TAAR1 in neuromodulation deserves critical analysis.

In particular, we will review the literature about TAAR1 distribution in brain, its activation by endogenous compounds and psychoactive drugs, and its interaction with signal transduction pathways triggered by established neuromediators. The CNS effects observed after the administration of TAAR1 ligands will be discussed, functional implications will be drawn from transgenic models, and the putative association of single nucleotide polymorphisms (SNPs) with psychiatric disease will be analyzed.

## TAAR Expression

**FIGURE 1:**
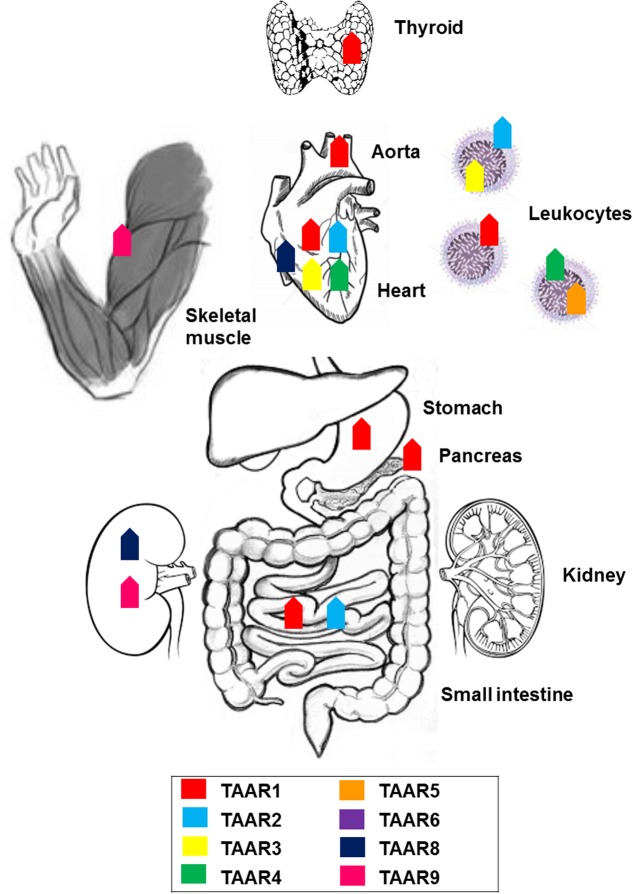
Anatomical distribution of trace amine-associated receptors (TAARs): expression of members of the TAAR family across the body.

**FIGURE 2:**
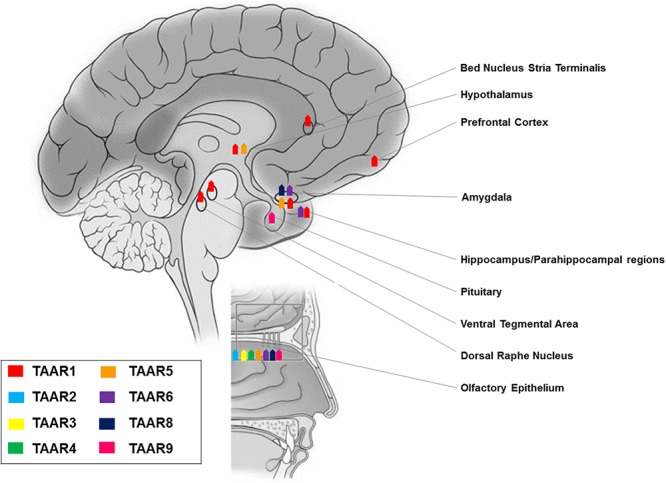
Anatomical distribution of TAARs: expression of members of the TAAR family in brain and olfactory mucosa.

The family of TAARs is widely distributed throughout peripheral and brain tissues. Quantitative reverse transcription (RT)-PCR revealed low levels (15–100 copies/ng cDNA) of TAAR9 mRNA in mouse kidney (Borowsky et al., 2001), and human skeletal muscle and pituitary (Vanti et al., 2003). TAAR8 was found in mouse kidney, mouse amygdala (Borowsky et al., 2001) and rat heart (Chiellini et al., 2007); TAAR2, TAAR3, and TAAR4 could also be detected in the rat heart, even if at substantially lower level (Chiellini et al., 2007). Expression of TAAR2, TAAR3, and TAAR4, together with TAAR5, was additionally reported in rodent and human peripheral leukocytes, and particularly in B cells and NK cells, but not in cultured macrophages or dendritic cells (D’Andrea et al., 2003; Nelson et al., 2007). TAAR6 has been found at low levels in mouse amygdala and hippocampus (Borowsky et al., 2001). Through *in situ* hybridization, signals specific for TAAR5 were observed in the mouse amygdala and hypothalamic regions involved in the regulation of weight and body temperature, namely the arcuate nucleus and the ventromedial hypothalamus (Dinter et al., 2015) (**Figures 1**, **2**).

Studies in rodent, primate, and fish elucidated a chemosensory olfactory function for all TAARs, except TAAR1 (Liberles and Buck, 2006; Hussain et al., 2009; Horowitz et al., 2014). TAARs are expressed in the mouse olfactory epithelium at levels overlapping those of odorant receptor genes (Liberles and Buck, 2006), and in the neonatal Grueneberg ganglion (Fleischer et al., 2007), but not in the vomeronasal organ (Liberles and Buck, 2006). Distinct TAARs define unique sensory neuron populations, as they co-localize neither with other TAARs nor with odorant receptors (Liberles and Buck, 2006). In olfactory neurons, TAARs are localized in cilia, the site of odor detection, and in axons (Johnson et al., 2012). TAAR-expressing neurons project to discrete glomeruli (Johnson et al., 2012) and sense volatile amines, some of which may act as aversive or attractive social cues (Liberles, 2015). Notably, evidence of TAAR5 expression in olfactory mucosa has also been reported in human (Carnicelli et al., 2010) (**Figure 2**).

Within the TAAR family, a unique characteristic of TAAR1 is the absence from the olfactory system of rodent, primate, and fish (Liberles and Buck, 2006; Hussain et al., 2009; Horowitz et al., 2014). On the other hand, its mRNA was detected in rodents at moderate levels (100 copies/ng cDNA) in stomach, at low levels in small intestine, and at trace (<15) levels in pancreas (Borowsky et al., 2001; Bunzow et al., 2001). TAAR1 gene transcripts were, jointly with TAAR2, the most abundant in the mucosal layer of the duodenum in mice (Ito et al., 2009). Histological data provided confirmation of the presence of TAAR1 in the gastrointestinal tract and in the insulin-secreting β cells, but not the glucagon-secreting α cells, of human and mouse pancreatic Langerhans islets (Raab et al., 2016). Therefore, TAAR1 appears to be substantially expressed in organs responsible for food absorption and regulation of glucose metabolism. Trace levels of TAAR1 were detected in the cardiovascular system, both in the rat heart (Bunzow et al., 2001), and aorta (by RT-PCR and by Western blotting), where it could mediate trace amine-induced vasoconstriction and elevation of blood pressure (Fehler et al., 2010). TAAR1 gene transcripts were, jointly with TAAR2, the most abundant in human polymorphonucleates and lymphocytes, to suggest a potential role in immune functions (Babusyte et al., 2013). Using immunofluorescence microscopy and immunoblotting, TAAR1 was found in lumen-apposed apical plasma membrane domains and in reticular and vesicular structures in the cytoplasm of thyroid follicle cells in mice, as a suggested target of thyronamines in a non-classical mechanism of thyroid autoregulation (Szumska et al., 2015). The initial reports of other peripheral tissues, namely kidney, lung, liver, prostate, testis, skeletal muscle, and spleen harboring TAAR1 at trace to low levels (Borowsky et al., 2001; Bunzow et al., 2001; Chiellini et al., 2012), have not been confirmed by recent analysis using more specific TAAR1 antibodies (Revel et al., 2013; Raab et al., 2016) (**Figure 1**).

RT-PCR experiments revealed TAAR1 expression in many distinct rodent CNS regions, namely olfactory bulb, nucleus accumbens/olfactory tubercle, hypothalamus, pituitary, cerebellum, pontine reticular formation, and most intriguingly the prefrontal cortex and other cortical areas, as well as limbic and monoaminergic areas, such as hippocampus, amygdala, substantia nigra, and ventral tegmental area (Borowsky et al., 2001; Bunzow et al., 2001). These results were confirmed and further detailed by *in situ* hybridization histochemistry, which showed: intense staining in mitral cell layer of the olfactory bulb, piriform cortex, arcuate, motor, and mesencephalic trigeminal nuclei, lateral reticular and hypoglossal nuclei, cerebellar Purkinje cells, and ventral horn of the spinal cord; moderate labeling in frontal, enthorinal, and agranular cortices, ventral pallidum, thalamus, hippocampus, hypothalamus, ambiguous, gigantocellular reticular nuclei, dorsal raphe nucleus, locus caeruleus, and ventral tegmental area; weak labeling in septum, basal ganglia, amygdala, myelencephalon, and dorsal horn of the spinal cord (Borowsky et al., 2001).

However, replacing the entire TAAR1 coding sequence with a reporter gene consisting of LacZ fused to a nuclear localization sequence to analyze TAAR1 tissue distribution, left some of the above reported areas unrecognized, presumably because of the lower sensitivity of this approach as compared to *in situ* hybridization (Lindemann et al., 2008). Notably, this TAAR1 knockout mouse line consistently allowed the identification of TAAR1 in: hypothalamus and preoptic area, known to modulate sleep (Chung et al., 2017) and energy expenditure (Coborn et al., 2017); ventral tegmental area, a dopaminergic area critical for learning processes and motivated and addictive behaviors (Langlois and Nugent, 2017); amygdala, a complex structure with a broad array of actions in emotional – especially fear – processing, reward learning and motivation, aggressive, maternal, sexual, and ingestive behaviors, and cognitive functions (LeDoux, 2007); dorsal raphe nucleus, a serotonergic region involved in cognition, reward, pain sensitivity, and circadian rhythms (Zhao et al., 2015); bed nucleus of the stria terminalis, relevant for the control of autonomic, neuroendocrine and behavioral – defensive and reproductive – responses (Crestani et al., 2013); parahippocampal region and subiculum, which play a fundamental role in memory processes (O’Mara et al., 2001; Lindemann et al., 2008; Pilly and Grossberg, 2013). These findings have been generalized to primates, as a wide distribution of TAAR1 mRNA and proteins was demonstrated in rhesus monkey brain monoaminergic nuclei (Xie et al., 2007).

**FIGURE 3:**
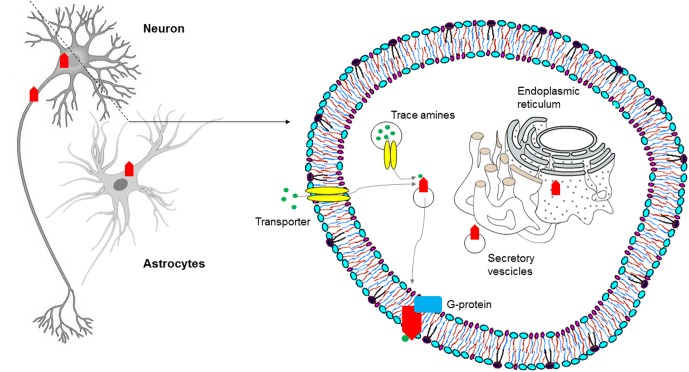
Subcellular distribution of TAARs: intracellular localization of TAAR1 in the central nervous system (CNS).

More recently, on the basis of pharmacologic studies indicating pro-cognitive actions of TAAR1 agonists (Revel et al., 2013), growing interest has risen as to whether TAAR1 is expressed and has a role in the prefrontal cortex, a brain region with prominent cognitive functions. Using a double approach – RT-PCR and histoenzymology – TAAR1 expression could be detected in the frontal cortex of mice, in addition to the aforementioned monoaminergic areas (Di Cara et al., 2011). More in detail, TAAR1 mRNA and fluorescent signal were consistently found in layer V cortical neurons in rodent prefrontal cortex (Espinoza et al., 2015b) (**Figure 2**). Besides neurons, TAAR1 was found to be expressed in the cytoplasm and nucleus of human astrocytes by means of RT-PCR and immunocytochemistry/confocal microscopy (Cisneros and Ghorpade, 2014) (**Figure 3**).

In order to determine the physiological role of TAAR1, several investigations have focused on its coupling with a second messenger system. In molecular pharmacology, this is commonly obtained for GPCRs through immortalized, clonal cell lines stably expressing heterologous receptors. However, repeated attempts to reliably express TAAR1 and to identify its messenger system(s) proved disappointing, a potential obstacle being the prominent intracellular localization of the receptor, as shown by confocal images of HEK293 cells expressing an engineered rat TAAR1 carrying an epitope tag at the N-terminus (Bunzow et al., 2001). Experiments with transient expression of TAAR1 fared better, notwithstanding the low signal to noise ratios and variable receptor densities of those preparations (Grandy, 2007), and similarly showed a predominantly intracellular distribution of TAAR1, with rare instances of membrane expression (Miller et al., 2005). Moreover, the use of cell fractionation techniques combined with biotinylation and Western blotting demonstrated the association of rhesus monkey TAAR1 with membrane fraction, but not with the extracellular one (Xie et al., 2008a). The receptor’s localization *in vivo* seems to recapitulate such findings, in that both *in situ* hybridization histochemistry in mouse and immunohistochemical analysis in rhesus monkey revealed a largely cytoplasmatic signal in neurons, as punctate foci within the perikaryon extending into the axon, with rare membrane-associated expression (Borowsky et al., 2001; Xie et al., 2007). TAAR1 lacks N-terminal glycosylation sites (Barak et al., 2008), and this might be the reason why it mainly remains intracellular, in the endoplasmatic reticulum or in vesicular membranes. In this location it could act as a binding site for agonists synthesized in the cytoplasm of trace amine-producing cells. Possibly, agonists could be transferred to the cytoplasm and/or vesicular lumen by plasma membrane and vesicular transporters (Bunzow et al., 2001). Alternatively, an accessory protein, most probably another GPCR, may be needed for TAAR1 to dynamically translocate to the plasma membrane in response to transporter-mediated agonist uptake (Xie et al., 2007; Espinoza et al., 2011; Harmeier et al., 2015) (**Figure 3**). It has been proposed that the binding of trace amines to the intracellular TAAR1 may favor heterodimerization with dopamine D2 receptors and translocation to the plasma membrane, as discussed below.

## Receptor Pharmacology

The early pharmacological characterization of TAAR1 was significantly slowed down by the difficulties in ensuring a stable *in vitro* expression in the plasma membrane. Indeed, transient transfection for TAAR1 in heterologous cell lines led either to TAAR1 degradation or to intracellular sequestration (Borowsky et al., 2001; Bunzow et al., 2001; Miller et al., 2005; Barak et al., 2008), and, therefore, to the impossibility to test its response to putative ligands. This obstacle was creatively overcome by several groups, who employed different approaches: co-expression of TAAR1 with rat Gαs (Wainscott et al., 2007) or Gq-Gα16 signaling proteins (Navarro et al., 2006); modification of receptor N-terminus, in order to ensure its membrane expression (Barak et al., 2008); creation of rat and human chimeras (Lindemann et al., 2005; Reese et al., 2007). Since exposure to TAAR1 agonists induced the activation of intracellular pathways that ultimately lead to cAMP synthesis, in most investigations TAAR1 activation was evaluated on the basis of cAMP production. In the systems co-expressing TAAR1 and Gq-Gα16 signaling proteins, TAAR1 activation was coupled to the mobilization of intracellular calcium (Navarro et al., 2006).

**FIGURE 4:**
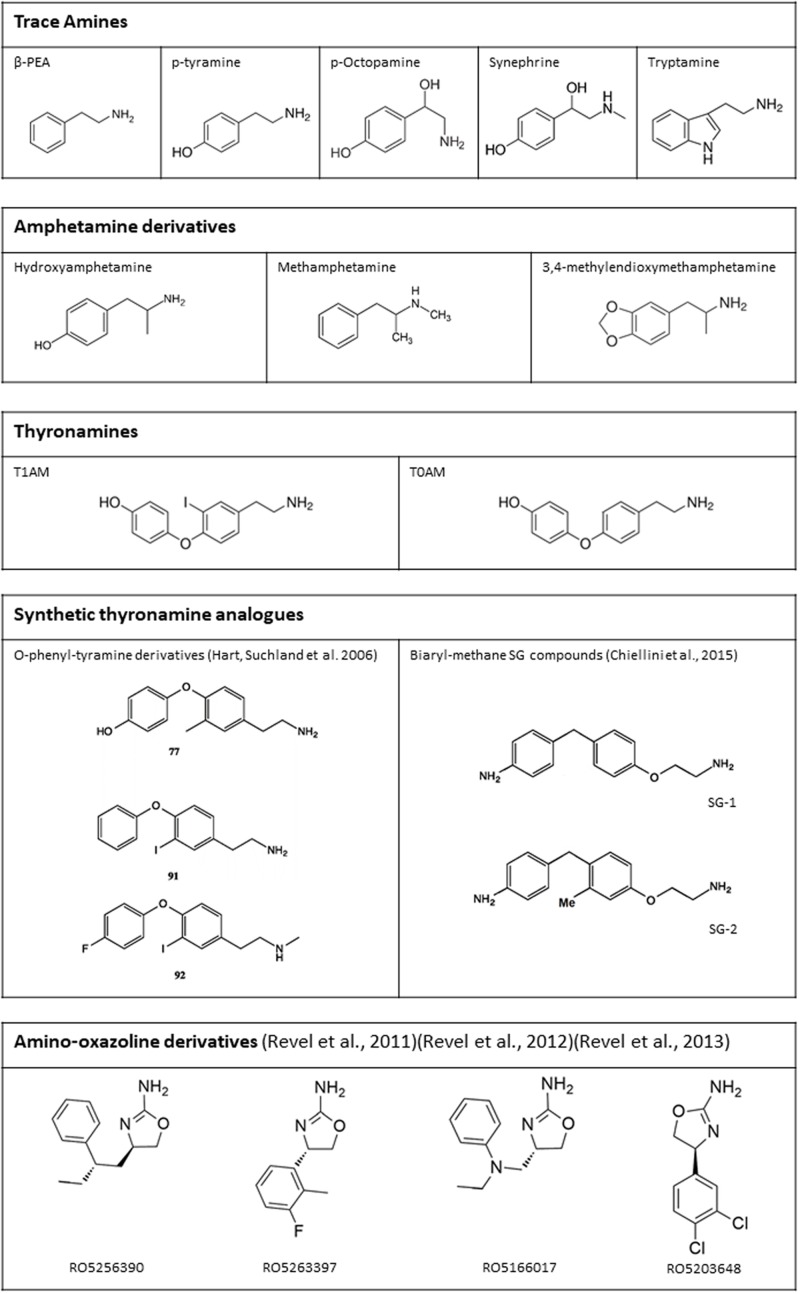
Chemical structure of some endogenous and synthetic TAAR1 ligands. Additional drugs which have been reported to act on TAAR1 are mentioned in the text (receptor pharmacology).

Once a stable extracellular membrane expression was ensured, and the specific reporting system identified, TAAR1 ligands have been extensively characterized (**Figure 4**). TAAR1 owes its name to the fact that, in the first two studies that led to its identification, trace amines, particularly PEA and TYR, were more potent than catecholamines and serotonin in activating the receptor (Borowsky et al., 2001; Bunzow et al., 2001). Indeed, the EC_50_’s of trace amines were in the nanomolar range, whereas dopamine, norepinephrine, epinephrine, and serotonin had EC_50_’s in the micromolar range. According to Bunzow et al. (2001), TYR was more potent than PEA in stimulating rat TAAR1 (EC_50_ 69 ± 9 and 240 ± 71 nM respectively). Whereas, Borowsky et al. (2001) could not find any significant differences in potencies of the two compounds on human TAAR1. Bunzow’s finding was subsequently confirmed by Reese et al. (2007), while Borowsky’s result was not corroborated by later studies (Lindemann et al., 2005; Navarro et al., 2006; Reese et al., 2007; Wainscott et al., 2007) that demonstrated that PEA shows the highest potency in human TAAR1 activation. In general, the affinity for the single trace amines was higher in rat than in mouse and human, and the difference between human and rat TAAR1 often exceeded one order of magnitude (reviewed by Berry et al., 2017).

The study by Bunzow et al. (2001) also represented the first systematic and extensive characterization of TAAR1 pharmacology. Indeed, by using HEK293 cells, stably transfected with rat TAAR1, they screened a large number of compounds. Their evaluation of the differential responses to trace amines and catecholamines, and the ranking of potencies of the different compounds, revealed that the presence of a hydroxyl group at the 3-position of the PEA molecule or at the 5-position of the tryptamine molecule significantly reduced agonist potency. The structural explanation of this finding is probably the lack of two serine residues, which are present in the ligand binding pocket of adrenergic and serotonergic receptors, and form a hydrogen bond with the ligand meta-hydroxyl group. In TAAR1, these serines have been replaced by alanine and phenylalanine, respectively (Grandy, 2007). Another interesting finding was the observation that the *O*-methyl derivatives of dopamine, norepinephrine, and epinephrine were more potent than the parent compounds. Since these derivatives are physiologically produced by the enzyme catechol-*O*-methyl transferase (COMT), and TAAR1 is present in areas where COMT expression is demonstrated to be highest (Mannisto and Kaakkola, 1999), a potential physiological role of TAAR1 in modulating adrenergic systems was hypothesized, as discussed below.

Since PEA and TYR share the same phenylethylamine structure with amphetamines, Bunzow et al. (2001) inquired if these drugs of abuse could activate TAAR1, obtaining positive results (Bunzow et al., 2001). The affinity for rat TAAR1, as evaluated on the basis of EC_50_’s, was in the micromolar range. *p*-Hydroxyamphetamine was the most potent derivative, whereas *N*-ethyl analogs had significant lower activities when compared to amphetamine and its *N*-methyl derivatives [methamphetamine and 3,4-methylenedioxymethamphetamine (MDMA)]. The latter result was confirmed in rhesus monkey TAAR1 (Miller et al., 2005). Later studies confirmed these findings and, at the same time, unraveled that TAAR1 from different species showed different stereospecificity to amphetamine and its derivatives (Reese et al., 2007; Wainscott et al., 2007; Lewin et al., 2008; Simmler et al., 2016).

Other abuse substances and psychoactive drugs were found to activate TAAR1, with EC_50_ in the nanomolar to micromolar range. These include lysergic acid diethylamide, bromocriptine, lisuride, nomifensine, apomorphine, ractopamin, clonidine, guanabenz, idozoxan, aminoindanes (2-aminoindane and 5-iodo-2-aminoindane), and *m*-chlorophenylpiperazine (Bunzow et al., 2001; Hu et al., 2009; Liu et al., 2014; Sukhanov et al., 2014; Simmler et al., 2016).

Aside trace amines, another class of endogenous amines able to interact with TAAR1 is represented by thyronamines. 3-iodothyronamine (T1AM) is an endogenous compound, detected in most rodent tissues and in human blood, probably derived from thyroid hormone through deiodination and decarboxylation (reviewed by Hoefig et al., 2016). Scanlan et al. (2004) reported T1AM to be the most potent endogenous TAAR1 agonist, with an EC_50_ for rat TAAR1 of 14 nM. As observed for trace amines, the affinity for mouse and human TAAR1 was one to two orders of magnitude lower (Coster et al., 2015). Other thyronamines, particularly 3,5-diiodothyronamine, 3-3′-didiodothyronamine, 3,5,3′-triiodothyronamine, and thyronamine (T0AM), were also able to activate TAAR1, but they were at least 5 to 10-fold less potent than T1AM (Scanlan et al., 2004). T0AM is also an endogenous compound, while the other thyronamines have not been detected in tissues so far (Saba et al., 2010). Although the development of an analytical assay for T1AM is still an open question, plasma and tissue T1AM concentration is probably higher than the conventional limit of 100 ng/g (Saba et al., 2010; Hoefig et al., 2011, 2016; Galli et al., 2012). In any case, thyronamines are not usually included in the group of trace amines.

TYR, PEA, and T1AM are the most likely candidates as physiological TAAR1 agonists. However, it should be kept in mind that they all share additional molecular targets. PEA and TYR have long been known to increase catecholamine and serotonin availability by competing with their receptors, transporters, or storage sites. However, given the fact that monoaminergic transporter activity is dependent on ligand concentrations (Yatin et al., 2002), the low tissue concentrations of trace amines casts doubts on their functional role. On the other hand, T1AM has been reported to be a multitarget ligand, since it can also interact with other TAAR subtypes (particularly TAAR5), α2A- and β-adrenergic receptors, TRM8 calcium channels, and membrane amine transporters like dopamine transporter (DAT), norepinephrine transporter (NET), and vesicular monoamine transporter (VMAT) (reviewed by Hoefig et al., 2016). The affinity for these additional targets is substantially lower than the affinity for rat TAAR1, but the difficulties in assessing T1AM concentration at receptor level do not enable to reach a clear conclusion about their physiological relevance. As discussed below, the presence of non-TAAR targets is an important pitfall in the interpretation of experimental results obtained with the administration of natural TAAR1 ligands.

Considerable effort has been devoted to the development of synthetic TAAR1 agonists. After the discovery that T1AM is a TAAR1 agonist, two series of T1AM analogs (mostly phenyltyramine derivatives: see **Figure 4** for the structure of some of the most active compounds) were synthesized and tested on the basis of cAMP production in heterologous cells expressing mouse or rat TAAR1 (Hart et al., 2006; Tan et al., 2007). These investigations established some milestones for the analysis of thyronamine structure-activity relationship (reviewed by Chiellini et al., 2017), and showed that the thyronamine scaffold is amenable to several types of chemical modifications, which can preserve or even increase the activity of the parent compound. More recently, another class of halogen-free biaryl-methane thyronamine analogs was obtained and tested both *in vitro* and *in vivo* (Chiellini et al., 2015, 2016). The analogs known as SG1 and SG2 showed similar potency as the endogenous ligands, and some SG2 derivatives were even more potent. While these compounds are certainly TAAR1 agonists, their selectivity has not been extensively evaluated yet, and they share with T1AM some functional effects (e.g., stimulation of hepatic gluconeogenesis) which may not be TAAR1-mediated (Regard et al., 2007).

Another approach was followed by the investigators at Hoffmann-La Roche, who produced an iterative series of structural modifications on adrenergic ligands, including the amino-oxazoline α2A-adrenergic receptor agonist S18616. In this way a potent full TAAR1 agonist, RO5166017, was obtained (Revel et al., 2011). Other full agonists (e.g., RO5256390) and partial agonists (e.g., RO5203648 and RO5263397) were subsequently identified (Revel et al., 2012, 2013). The “RO compounds” were reported to be highly selective for TAAR1 on the basis of a screening procedure based on radioligand binding assays involving over 100 target proteins. They were therefore widely used in functional experiments to determine the effects of TAAR1 stimulation, as reviewed below. However, it should be pointed out that micromolar concentration of these compounds produced >80% inhibition of specific ligand binding at other receptors, namely some subtypes of adrenergic (α2), serotonergic (5-HT2A and 5-HT3), opioid (especially κ and μ), imidazoline (especially I1), and muscarinic receptors. The selectivity ratio vs. TAAR1 was usually >100, but Ki’s were nevertheless in the nanomolar range (Revel et al., 2011).

A strong effort was also made to identify TAAR1 antagonists. To this purpose, over 700,000 Roche compounds were screened on the basis of the capacity to inhibit cAMP production triggered by PEA in cells expressing a chimeric human/rat TAAR1 receptor (Bradaia et al., 2009; Stalder et al., 2011). A benzamine derivative [RO5212773 or EPPTB, *N*-(3-Ethoxy-phenyl)-4-pyrrolidin-1-yl-3-trifluoromethyl-benzamide (EPPTB) (Galley et al., 2009)] was eventually selected. It shows good selectivity and very high affinity for mouse TAAR1 (Ki = 0.9 nM), with lower affinity for rat TAAR1 (Ki = 942 nM) and human TAAR1 (Ki > 5 μM). Interestingly, basal cAMP levels were reduced by EPPTB, suggesting that TAAR1 may be constitutively active and that EPPTB should be regarded as an inverse agonist, rather than as a neutral antagonist (Bradaia et al., 2009). However, it has not been formally excluded that endogenous agonists may have been present in the preparations used in this investigations. Notably, EPPTB has a high clearance, which limits its use *in vivo* (Stalder et al., 2011; Berry et al., 2017).

## Cross Talk Between TAAR1 and Monoaminergic Systems

Consistent with its CNS distribution, TAAR1 appears to interact with other monoaminergic systems. *In vivo*, the use of TAAR1 agonists lowered hyperlocomotion in pharmacologic, i.e., cocaine-induced, and genetic, i.e., DAT-knockout (KO), models of hyperdopaminergia (Revel et al., 2011, 2013), allegedly a hallmark of psychosis (Howes and Kapur, 2009). Intracellular electrophysiological recordings showed significant decrease in spontaneous firing rate and membrane hyperpolarization under application of TYR (10–100 μM) (Geracitano et al., 2004; Lindemann et al., 2008), PEA (Geracitano et al., 2004), or the synthetic TAAR1 agonist RO5166017 (500 nM) in mouse and rat dopaminergic and serotoninergic neurons, respectively from the ventral tegmental area and the substantia nigra pars compacta, and from the dorsal raphe nucleus (Revel et al., 2011). These effects were counteracted by the application of EPPTB (10 nM) under current-clamp conditions (Bradaia et al., 2009). Moreover, when EPPTB was applied alone, the basal firing rate of dopaminergic neurons was significantly and reversibly enhanced, as discussed above (Bradaia et al., 2009).

Converging evidences point to K^+^ currents as effectors of membrane hyper/de-polarization effects respectively induced by TYR or EPPTB. Firstly, in voltage-clamp conditions, dopaminergic neurons responded to TYR with a drop in input resistance (Geracitano et al., 2004; Bradaia et al., 2009). By applying voltage ramps from -20 to -140 mV, dopaminergic neurons stimulated with TYR at physiological extracellular [K^+^] (2.5 mM) exhibited an inwardly rectifying current whose polarity reversed close to the calculated K^+^ equilibrium potential (-101 mV), and which was sensitive to EPPTB (Bradaia et al., 2009). The reversal potential of the induced current was shifted to -60 mV upon alteration of extracellular [K^+^] to 12.5 mM (Bradaia et al., 2009). The current could be abolished by the non-selective K^+^ channel blocker Ba^2+^ (300 μM) and the Kir3 channel blocker tertiapin (10 μM), but not in presence of protein kinase A and mixed Na^+^/K^+^ current inhibitors (Bradaia et al., 2009). Since blocking G protein activation with GDPβS attenuated the current, it is plausible that TAAR1 gates Kir3-type K^+^ channels through the Gβγ subunits (Bradaia et al., 2009), as already known for other GPCRs (Mark and Herlitze, 2000).

The electrophysiological effects of stimulating TAAR1 might involve either monoamine autoreceptors or transporters. Monoamine autoreceptors function as presynaptic feedback regulators for monoamine release. The effects of trace amines were extinguished by the application of dopamine autoreceptors (D2R) antagonists, such as sulpiride, as well as by treatment with reserpine, which depletes presynaptic dopamine stores, in combination with carbidopa, a dopa decarboxylase inhibitor. Therefore, it was initially proposed that the inhibitory effects of PEA and TYR could be mediated by indirect stimulation of D2R via an increase of dopamine release (Geracitano et al., 2004). The final effect might be a tonic enhancement of D2R-related autoinhibition by TAAR1 activation, as part of a rheostatic mechanism regulating the activity of dopaminergic neurons (Leo et al., 2014). However, RO5166017 (10 μM) was shown to decrease dopamine release in both the dorsal striatum and the nucleus accumbens, while EPPTB (10 μM) evoked an increase of dopamine overflow selectively in nucleus accumbens, a brain area which receives projections from the ventral tegmental area (Leo et al., 2014). In addition, it has been reported that TAAR1 antagonists evoked a fourfold increase in agonist affinity to D2R and prevented D2R desensitization (Bradaia et al., 2009). Evidence exists that stimulating TAAR1 with PEA (1 μM) significantly reduces D2R membrane expression, in support of a mechanism of receptor internalization underpinning its desensitization (Espinoza et al., 2011). On the contrary, TAAR1 antagonists decreased the potency of ipsapirone at serotonergic autoreceptors (5-HT1A), and abolished 5-HT1A desensitization (Revel et al., 2011).

Reciprocally, monoaminergic receptors were found to modulate TAAR1 activity. In HEK293 cells co-transfected with TAAR1 and autoreceptors (D2R, 5-HT1A/1B, and α2A/2B), the response to PEA and common biogenic amines was lower than in TAAR1-expressing cells, and was enhanced by means of selective D2R antagonists (Xie et al., 2007, 2008b; Espinoza et al., 2011; Harmeier et al., 2015). Controversial findings apply as to whether trace amines directly modulate autoreceptors, which in turn attenuated TAAR1 signaling (Xie and Miller, 2008), or rather co-transfection with D2R, 5-HT1A/1B, and α2A/2B reduced TAAR1 expression (Xie and Miller, 2008; Espinoza et al., 2011). An alternative possibility is that monoamine autoreceptors can affect AADC activity and therefore trace amine synthesis (Berry, 2004).

Experiments with either bioluminescence resonance energy transfer measurement assays or co-immunoprecipitation demonstrated that TAAR1 and D2R specifically interact to form heterodimers, mainly at the level of plasma membrane (Espinoza et al., 2011; Harmeier et al., 2015). One could speculate that, after crossing the plasma membrane, trace amines may bind to and induce a conformational change in intracellular TAAR1, which then translocates to the plasma membrane to form heterodimers with biogenic amine receptors (Berry, 2004). Following heteromerization, TAAR1 signals through Gαs to increase cAMP levels (Borowsky et al., 2001; Bunzow et al., 2001), while D2R signals through Gαi to decrease cAMP levels (Lindemann et al., 2008). Upon TAAR1-D2R heteromerization, cAMP accumulation after activation of TAAR1 was decreased, that led to reduced phosphorylation of the downstream effector proteins ERK1/2 and CREB (Harmeier et al., 2015). On the other hand, TAAR1 stimulation in presence of D2R triggered β-arrestin2 recruitment, and downstream silencing of the GSK3β cascade via Akt and GSK3β (Harmeier et al., 2015). Interestingly, the latter pathways play a role in psychosis and mood disorders (Willi and Schwab, 2013), and are targets of lithium (Muneer, 2017).

As TAAR1 localization is mainly intracellular (see above), it has been hypothesized that monoamine transporters could provide conduits for trace amines, to cross the plasma membrane and act on their molecular target (Miller, 2011). In favor of this hypothesis, both RT-PCR and fluorescence microscopy on rhesus monkey and mouse brain sections allowed the co-detection of signals from TAAR1 and DAT in dopaminergic neurons in the substantia nigra, amongst neurons expressing TAAR1 or DAT alone (Miller et al., 2005; Xie et al., 2007). Indirect evidence suggests co-expression of TAAR1 with DAT and the serotonin transporter (SERT) mainly in the striatum, and with the norepinephrine transporter (NET) in the thalamus (Xie et al., 2008b). As expected, co-transfecting HEK293 with TAAR1 and DAT, SERT, or NET potentiated TAAR1 signaling (Miller et al., 2005; Xie et al., 2007). TAAR1 was also demonstrated to respond to common biogenic amines, such as dopamine, serotonin, and norepinephrine (Xie et al., 2008b). These effects were counteracted by DAT, NET, and SERT inhibitors (Xie et al., 2007). A synergic relationship might link TAAR1 and monoamine transporters, as several TAAR1 agonists are substrates for monoamine transporters, as well. However, trace amines appear to be substrates of DAT, SERT, and NET at high micromolar, or millimolar concentrations, which are not expected under physiological conditions (Berry, 2004). On the contrary, no potentiation of TAAR1 activity could be observed with T1AM (Scanlan et al., 2004), which acts as inhibitor, rather than substrate, of DAT (Panas et al., 2010). Recently, the Organic Cation Transporter 2 (OCT2) has been identified as a high affinity neuronal transporter for trace amines at physiologically relevant concentrations (Berry et al., 2016). The OCT family is known as a polyspecific, low-selectivity, high capacity family of transporters which mediate the clearance of monoamines when DAT, SERT, and NET become saturated (Courousse and Gautron, 2015). Given such functional link, neurons expressing both TAAR1 and monoamine transporters could be preferentially activated by pharmacological agonists, e.g., amphetamines, thus contributing a crucial role in the development of addiction to amphetamine-like drugs of abuse (Miller, 2011).

**FIGURE 5:**
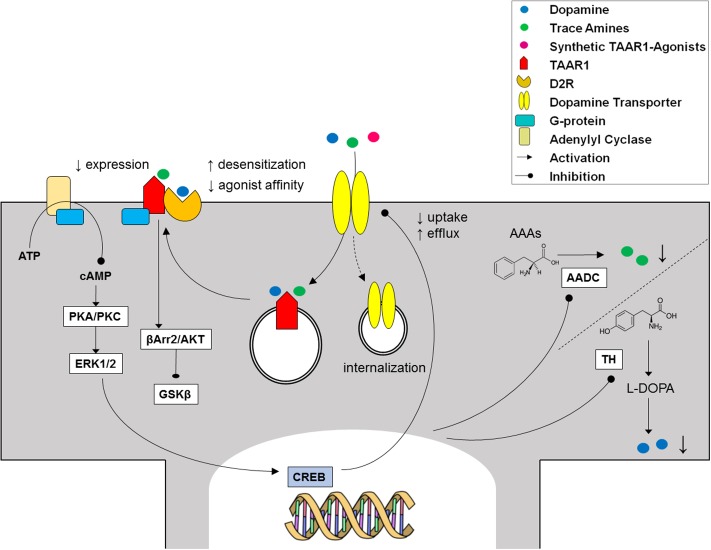
Cross-talk between TAAR1 and the dopaminergic system.

Evidence of a reciprocal regulation of monoamine transporters by TAAR1 came from *in vitro* experiments, where it was found that pretreatment with dopamine, serotonin, NE, and methamphetamine significantly inhibited monoamine uptake in HEK293 cells co-expressing TAAR1 and DAT/SERT/NET (Xie and Miller, 2007, 2009), a finding that was later confirmed in synaptosomes (Xie et al., 2008b; Xie and Miller, 2009). This effect was potentiated by pretreatment with the selective monoamine autoreceptor inhibitors, to suggest that concurrent activation of autoreceptors from biogenic amines may act as a brake on TAAR1 influence on monoamine transporters (Xie et al., 2008b). Furthermore, treatment with methamphetamine triggered the internalization of DAT in TAAR1-DAT cells and in wild-type mice striatal synaptosomes (Xie and Miller, 2009). The observed regulatory actions of TAAR1 on transporters are supposedly dependent on cAMP accumulation and PKC-phosphorylation, as they were prevented by the PKC inhibitor Ro32-0432 (Xie and Miller, 2007, 2009; Xie et al., 2008b). Therefore, common biogenic amines released into the synaptic cleft could interact in parallel with monoamine autoreceptors and TAAR1 to modulate their own release and transport in the brain. Additional regulation of TAAR1 may come from trace amines, which share spatial distribution with the monoaminergic systems (Berry, 2004) and were similarly found to inhibit uptake and promote efflux by monoamine transporters (Xie and Miller, 2008). However, TAAR1-KO and wild-type mice showed overlapping dopamine uptake and half-life, indicating normal DAT activity (Leo et al., 2014), consistent with unaltered *in vivo* functional activity of TAAR1 agonists over the behavioral abnormalities of DAT-KO mice (Giros et al., 1996; Sotnikova et al., 2004; Revel et al., 2012). On the whole, the relevance of TAAR1 interaction with brain monoamine transporters still awaits clarification (**Figure 5**).

Recently, novel evidence has emerged about the role of TAAR1 as a modulator of glutamatergic transmission in the prefrontal cortex. TAAR1 agonists were able to suppress the hyperlocomotion triggered by non-competitive NMDA receptor blockers, phencyclidine and L-687414, reminiscent of the antipsychotic drug olanzapine, but with no significant weight gain and catalepsy (Large, 2007; Revel et al., 2011, 2013). Also, TAAR1 activation decreased impulsivity in mice performing a classic Skinner’s schedule of reinforcement (Sagvolden et al., 1983; Espinoza et al., 2015b), and promoted pro-cognitive effects, mainly executive functions, in primates and rodents (Revel et al., 2013). Intriguingly, TAAR1 agonists affected brain perfusion, as imaged in pharmacological magnetic resonance imaging, with a similar albeit not identical pattern as olanzapine, namely activation of cortico-limbic areas and de-activation of more ventral, subcortical structures (Revel et al., 2013). TAAR1 modulation of the prefrontal cortex glutamatergic NMDA-related transmission could be indirect, through the interaction with the dopaminergic system. Alternatively, a direct communication might exist between TAAR1 and glutamate transmission. Consistently with the latter hypothesis, methamphetamine and TAAR1 overexpression significantly reduced the expression of excitatory amino acid transporter 2 in astrocytes, subsequently impairing the clearance of extracellular glutamate from the synaptic cleft (Cisneros and Ghorpade, 2014).

## Neural Effects of TAAR1 Ligands

As already indicated in the section on pharmacology, the first TAAR1 ligands to be identified were trace amines (**Table 1**). Initially, they were thought to be merely the catabolic products of the classical monoamines; however, subsequently, it has been demonstrated that they produce physiological and pathophysiological effects in their own right.

**Table 1:** Neural effects of endogenous TAAR1 agonists.

Agonist	Neural effect
*p*-Octopamine	Enhances both excitatory and depressant responses to noradrenaline (fronto-parietal cortex)
	No effects on serotoninergic and dopaminergic transmission
β-PEA	Potentiates neuronal responses to dopamine (rat caudate nucleus, cortex)
	Increases neuronal responses to norepinephrine (cortex)
	No effects on serotonin and GABA
*p*-Tyramine	Potentiates neuronal responses to dopamine (cortex and caudate nucleus)
	Increases the responses of neurons to norepinephrine (cortex)
	No effects on serotonin and GABA
T1AM	Orexigenic effect when administered at the level of the arcuate nucleus
	Biphasic effect on food intake when administered i.c.v.
	Reduction of non-REM sleep (i.c.v.)
	Prolearning and anti-amnestic effect (i.c.v.)

*For references see text*.

Given the structural similarities with catecholamines, trace amines have been implicated in the induction of some of the effects associated with catecholaminergic stimulation. For example, PEA, that structurally is similar to amphetamine, has been demonstrated to mimic some of amphetamine effects, namely it induces locomotor activation and the performance of stereotyped behavior associated with catecholamine assumption in both rodents and rhesus monkeys (Borison et al., 1977; Tinklenberg et al., 1978; Ortmann et al., 1984; Dourish, 1985; Barroso and Rodriguez, 1996). However, it has to be underscored that the concentrations that were used to produce those effects were around the micromolar range, several orders of magnitude above their concentration in serum and tissue. Indeed, trace amine concentrations in tissues have been demonstrated to be comprised between 0.1 and 10 nM, not far from their TAAR1 EC_50_ estimated *in vitro* (Berry, 2004; Zucchi et al., 2006). Therefore, it is possible that catecholaminergic effects were only pharmacological and that most of trace amine physiological effects are mediated by high- affinity receptors like TAAR1.

With regard to their physiological roles in the CNS, trace amines have been proved to act as neuromodulators, potentiating or inhibiting the effects of the neurotransmitters with which they are co-released in the synaptic cleft (Berry, 2004). Octopamine enhances both excitatory and depressant responses of frontoparietal cortex neurons to norepinephrine, while it does not produce any effects on serotonin and dopamine transmission in those cortical areas (Jones, 1982). However, it produces a depressant action on the firing activity of dopaminergic neurons of the substantia nigra pars compacta (Pinnock, 1983). The same effect was elicited in the substantia nigra and in the ventral tegmental area by TYR and PEA (Pinnock, 1983; Geracitano et al., 2004; Lindemann et al., 2008). Even though PEA and TYR dampen dopaminergic neuron firing, some reports suggest that PEA and TYR may potentiate neuronal responses to dopamine both in the rat caudate nucleus (Jones and Boulton, 1980; Paterson et al., 1991; Sato et al., 1997) and in the rat cortex (Jones and Boulton, 1980). In rat cortical neurons, both trace amines also increases neuronal responses to norepinephrine administration (Paterson, 1988; Paterson and Boulton, 1988). Of note, these effects were only obtained at pharmacological concentrations of trace amines. On the other hand, both PEA and TYR do not produce any significant effects in the transmission of serotonin (Paterson et al., 1990) and GABA (Jones and Boulton, 1980; Paterson, 1988; Berry et al., 1994; Federici et al., 2005).

As the dopaminergic system plays a fundamental role in the regulation of the rewarding properties of addictive drugs (Cooper et al., 2017), selective TAAR1 agonists and antagonists were used to investigate the putative role of TAAR1 in the field of addiction. The selective full and partial TAAR1 agonists RO5256390 and RO5203648 have been demonstrated to reduce cocaine peripheral (Pei et al., 2014, 2015) and intracranial self-administration (Pei et al., 2015) and the reinstatement of drug seeking behavior (Pei et al., 2014, 2015). Also, other TAAR1 partial and full agonists (RO5263397 and RO5166017) were proved to inhibit cocaine-conditioned place preference (Thorn et al., 2014; Liu et al., 2016).

Similar effects have been reported also for methamphetamine. Indeed, the administration of RO5203648 and RO5263397 reduced hyperlocomotion, self-administration and reinstatement of methamphetamine seeking behavior (Cotter et al., 2015; Pei et al., 2017). The mechanism underlying these effects might be represented by a modulation of addictive-drug induced release of dopamine. Indeed, several studies have corroborated the fact that TAAR1 agonists prevent drug-induced dopamine overflow and dopaminergic neuron increase in firing (Revel et al., 2011; Pei et al., 2017) in brain structures involved in addiction processes, such as the nucleus accumbens (Pei et al., 2017).

As discussed in the previous section, inhibition of an inwardly rectifying K^+^ current may be related to TAAR1-mediated modulation of dopaminergic neuros in the ventral tegmental area (Bradaia et al., 2009). Another mechanism that is thought to be involved in addictive behavior is the lack of control of impulsivity; this trait is typical of addiction, is likely due to a dysfunction of the prefrontal cortex (Bari and Robbins, 2013), and can also be reduced by full or partial TAAR1 agonists (Sagvolden et al., 1983; Espinoza et al., 2015b).

In addition, the use of TAAR1 agonists has started to uncover the possible role that TAAR1 may play in the development of diseases like schizophrenia, and Parkinson’s disease. Indeed, TAAR1 activation leads to increased vigilance in rodents and to pro-cognitive and antidepressant effects in both rodents and primate models (Revel et al., 2013). With regard to Parkinson’s disease, Alvarsson et al. (2015) have demonstrated that in 6-hydroxydopamine model of Parkinson’s disease, TAAR1 activation inhibits L-DOPA induced rotational sensitization and the development of L-DOPA -induced dyskinesias. Even though these results have only been demonstrated in rodents and the way to translate them into human studies is still very long, they open interesting perspectives in the understanding and possibly treatment of the two diseases.

Another endogenous class of compounds able to interact with TAAR1 is represented by thyronamines (**Table 1**). As reviewed in previous sections, T1AM activates TAAR1 with the highest affinity between tyronamines, and it has been demonstrated to produce relevant neurological effects. When analyzing the responses elicited by exogenous T1AM administration, it is important to consider the specific concentrations that have been obtained in the brain. In fact, baseline tissue T1AM levels lie in the nanomolar range (Hoefig et al., 2016), and the dosages used *in vivo* were found to increase them by only 20–30 times (Manni et al., 2013), suggesting a potential physiological role of endogenous T1AM.

T1AM modulates several CNS functions that include feeding behavior, sleep composition, learning and memory. With regard to the regulation of food intake, its administration at the level of the arcuate nucleus of mice fed *ad libitum* induces an orexigenic effect (Dhillo et al., 2009); whereas the administration in the cerebral ventricles (i.c.v.) has a biphasic effects, since, lower dosages (3.3 nmol/Kg) reduce food intake and higher ones (51 nmol/Kg) increase food intake in mice (Manni et al., 2012).

T1AM also modulates sleep pattern composition when administered i.c.v., reducing the duration of non-REM sleep in mice (James et al., 2013). T1AM has also been proposed as a novel memory enhancer. Indeed, its administration i.c.v., has been demonstrated to produce a pro-learning and anti-amnestic effect as assessed with the novel object recognition and the passive avoidance tests (Manni et al., 2013). Moreover, a recent study suggested that also T1AM metabolite 3-iodothyroacetic acid may be involved in the regulation of learning and memory processes (Laurino et al., 2015). Also, preliminary evidence suggests that T1AM may also have a neuroprotective role in Alzheimer’s disease, counteracting beta amyloid toxicity in a mouse model of Alzheimer’s disease both in an electrophysiological and a behavioral assessment (Accorroni et al., 2016).

However, as already underscored for trace amines, it should be considered that T1AM does not only bind to TAAR1 but can also interact with other receptors. Therefore, it is possible that other systems alongside with TAAR1 are involved in the induction of the effects that have been demonstrated in the literature.

## Transgenic Models and Human Genetic Investigations

In order to dissect TAAR1-mediated effects, TAAR1-KO mice were generated. Their phenotype appeared grossly normal, in terms of general health, viability, fertility, life span, nest building behavior, body size and weight, and body temperature. The examination of general motor functions did not reveal any difference in dexterity, motor coordination, and spontaneous locomotor activity. They obtained normal scores in neurological tests assessing sensory, motor and autonomic responses, visual acuity, grip strength, and nociception (Wolinsky et al., 2007; Lindemann et al., 2008; Di Cara et al., 2011). As for behavioral tests designed to mimic psychiatric symptoms, mice lacking TAAR1 showed no alteration in anxiety, stress response, and working memory, although significant impairment of sensorimotor gating functions could be observed (Wolinsky et al., 2007), which is postulated to be a hallmark of dopamine supersensitivity characteristic of positive psychotic symptoms (Thaker, 2008). On the same line, mutant mice displayed a perseverative and impulsive pattern of behavior (Espinoza et al., 2015b), and were generally slower in learning how to perform cognitive tests (Achat-Mendes et al., 2012; Espinoza et al., 2015b).

It is plausible that these behavioral phenotypes are underlined by alterations in the neural circuitry in monoaminergic systems. Accordingly, there is evidence of increased basal levels of extracellular dopamine selectively in the nucleus accumbens, but not the dorsal striatum, of TAAR1-KO mice (Leo et al., 2014). Of note, several previous studies had not been able to detect any difference in basal neurotransmitter concentrations in striatal regions (Wolinsky et al., 2007; Lindemann et al., 2008; Di Cara et al., 2011), most probably due to technical shortcomings of conventional microdialysis (Chefer et al., 2009). Electrophysiological recordings revealed signs of altered dopamine neurotransmission, namely higher spontaneous firing rates and depolarized resting membrane potential in dopaminergic neurons of the ventral tegmental area (Lindemann et al., 2008). Moreover, changes in D2R affinity state were detected in the striatum (Wolinsky et al., 2007), accompanied by D2R upregulation and enhanced D2R-mediated signaling (Espinoza et al., 2015a). In particular, in TAAR1-KO mice, Western blot and immunoprecipitation experiments showed a decreased phosphorylation state for AKT and GSKβ – two downstream targets of β-arrestin2-dependent pathway (Beaulieu et al., 2005; Beaulieu and Gainetdinov, 2011), with subsequent activation of GSKβ and degradation of β-catenin (Espinoza et al., 2015a). Dopamine hyper-activity is mirrored by the increased basal phosphorylation of tyrosine hydroxylase at Ser19, Ser31, and Ser40 (Haycock and Haycock, 1991; Di Cara et al., 2011). These data provide additional support to the hypothesis that TAAR1 may maintain a tonic negative control on dopamine neurotransmission in physiological conditions.

Following the evidence about the expression of TAAR1 in the prefrontal cortex, and the effects of the pharmacological manipulation of TAAR1 on glutamate neurotransmission, the role of TAAR1 in prefrontal cortex was further evaluated by electrophysiological approaches. In TAAR1-KO mice excitatory post-synaptic currents had increased amplitude and altered kinetics, due to NMDA deficiency and decreased NMDA/AMPA ratio (Espinoza et al., 2015b). The lack of TAAR1 modified NMDA composition, reducing the expression of NMDA GluN1 and GluN2B subunits, while no changes in the expression of NMDA GluN2A, AMPA GluA1 subunit, and PSD-95 (a post-synaptic protein crucial for the organization of post-synaptic structure and integrity) could be detected (Espinoza et al., 2015b). Furthermore, mice’s sensitivity to amphetamine, methamphetamine, MDMA, and ethanol was exacerbated. This emerged from behavioral tests, where transgenic animals resulted more susceptible to either the locomotor-stimulating or -depressing effects of various amphetamines (Lindemann et al., 2008; Di Cara et al., 2011; Achat-Mendes et al., 2012) or ethanol (Lynch et al., 2013), respectively. At neurochemical level, amphetamine challenge was associated to higher concentrations of dopamine, serotonin and NE in the striatum and prefrontal cortex (Wolinsky et al., 2007; Lindemann et al., 2008; Di Cara et al., 2011). Such sensitization effects of TAAR1 could account for its involvement in the rewarding effects of drugs of abuse. Accordingly, TAAR1-KO mice developed an earlier and longer-lasting methamphetamine-induced conditioned place preference as compared to wild-type littermates (Achat-Mendes et al., 2012).

**FIGURE 6:**
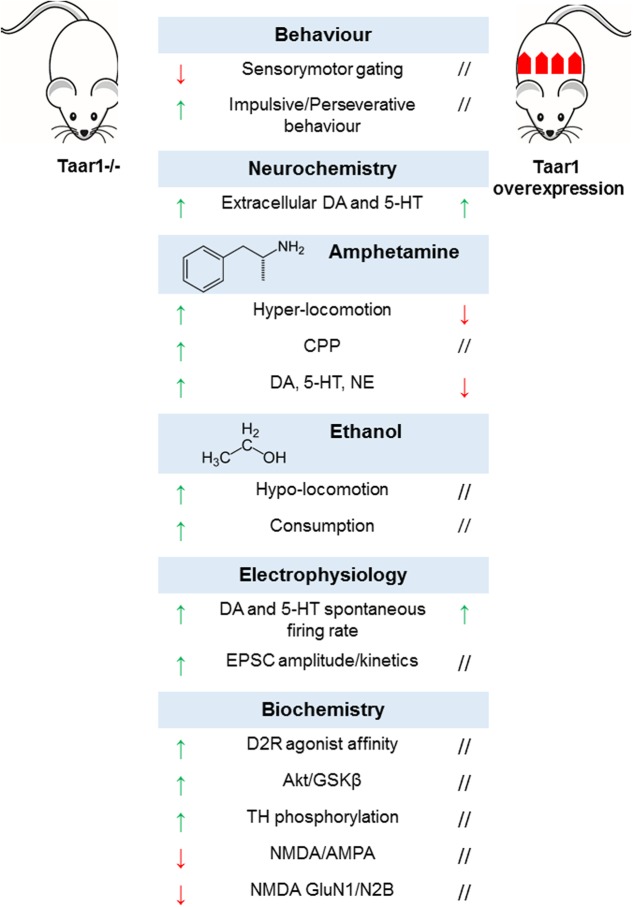
Multilevel effects of genetic manipulation of TAAR1 in murine models.

Similarly, TAAR1-KO genotype featured greater ethanol consumption, and resulted more sensitive to the sedative actions of ethanol, notwithstanding identical pharmacokinetic parameters (Lynch et al., 2013). Aside TAAR1-KO animals, the effects of TAAR1 overexpression were investigated in an *ad hoc* transgenic mouse line, where a Thy-1,2 expression cassette drove strong constitutive neuronal expression of TAAR1. As expected, mice overexpressing TAAR1 exhibited a lower response to the stimulating effects of amphetamine in terms of locomotor activity and monoamine release, opposite to TAAR1-KO mice. However, surprisingly, the effects of augmented TAAR1 expression paralleled those of TAAR1 deletion in some phenotypic aspects, including enhanced spontaneous electrical activity of dopaminergic neurons in the ventral tegmental area and serotonergic neurons in the dorsal raphe nucleus, associated to higher basal extracellular concentrations of dopamine and norepinephrine in the nucleus accumbens and of serotonin in the prefrontal cortex. To explain this inconsistency, it has been proposed that the ectopic expression of TAAR1 in GABAergic neurons in the ventral tegmental area could exert a tonic negative control on GABA firing activity, thus removing inhibitory inputs to dopaminergic neurons. Finally, TAAR1 overexpression did not trigger downregulation of monoamine receptors and transporters, despite altered monoamine levels (Revel et al., 2012). Taken together, these data suggest that TAAR1 might play a complex role in neuromodulation and contribute a novel target for the development of compounds aimed at treating neuropsychiatric disorders and substance abuse (Berry et al., 2017) (**Figure 6**).

The human and the chimpanzee genomes encompass nine TAAR genes. Rodent genomes include additional genes, probably originated by duplication events. All members of the TAAR family generate short transcripts made of short coding regions (∼1000 bp-long) with no introns, with the exception of the gene coding for TAAR2, which contains two exons. In mammals, TAARs cluster in a single chromosome. The human TAARs are located on chromosome 6 at band q23.1, a region that has been reported by several linkage and molecular genetic studies to be associated with mental disorders (Zucchi et al., 2006). As regards human TAAR1, about 600 SNPs have been identified, of which a couple of 100s are non-synonymous (dbSNP database, NCBI, accessed on the September 5, 2017). To elucidate their functional effects, Shi et al. (2016) transfected CHO-K1 cells with eight human TAAR1 constructs containing SNPs in highly conserved motifs, and measured receptor expression and cAMP accumulation upon PEA stimulation. They found that the substitution of a lysine (K218I) in the G protein-coupling region of the intracytoplasmic loop 3 altered receptor functionality, as compared to non-transfected cells, while the other selected variants were non-functional (Shi et al., 2016). Given the putative role of TAAR1, subjects carrying such sub-functional receptors might be predisposed to several mental disorders, including psychosis, mood disorders, attention-deficit/hyperactivity disorder (ADHD), and addiction. The use of whole exome sequencing allowed the identification of a heterozygous rare missense SNP (545G > T; C182F) in the affected mother and two affected siblings in a small schizophrenia family. *In silico* functional analyses predicted this variant to be damaging, as it causes the breakage of a highly conserved disulfide bond, with deleterious effects on receptor stability and cell surface localization, ligand binding, and G protein activation. Other six rare protein-disturbing variants (S47C, F51L, Y294T, L295S, A109T, V250A), all but one endorsing a potential for receptor damage, were found in sporadic patients suffering from schizophrenia of north Indian (*n* = 475) and American origin (*n* = 310), but in none of 410 healthy controls (John et al., 2017). The present data therefore suggest that TAAR1 may contribute an etiological role to the pathogenesis of schizophrenia, amongst a multiplicity of genetic and environmental risk factors.

## Conclusion

The bulk of evidence points to a significant physiological role of TAAR1 in the modulation of CNS function, and to a potential pharmacological role of TAAR1 agonists in neurology and/or psychiatry. This conclusion is based on the wide expression of TAAR1 in the CNS, the significant neurological and/or behavioral effects produced by the administration of natural or synthetic TAAR1 agonists, and the neurological phenotypes observed in TAAR1-KO mice.

However, there is still no formal demonstration that a specific endogenous mediator produces a specific effect in the CNS through TAAR1 stimulation. A major pitfall is the existence of additional targets for most, if not all, known TAAR1 ligands. Inhibition by EPPTB has been regarded as a criterion to attribute functional effects to TAAR1 stimulation. Although fairly specific for TAAR1, screening procedures based on the inhibition of standard ligand binding showed >80% inhibition at human A3 adenosine receptor and rat sodium channel, as well as >50% inhibition at human A1 adenosine receptor, human cholecystokinin 1 receptor, human melatonin 1 receptor, rat MAO-A, rat 5HT1B receptor, and rat GABA-dependent chloride channel (Stalder et al., 2011). Therefore, the responses to EPPTB should be confirmed through the use of another antagonist with a different molecular structure, which is not available at present.

Alternatively, the pharmacological investigations should be corroborated by convergent results obtained by molecular biology techniques. While TAAR1-KO animals have been produced and have provided interesting results, experimental models allowing conditional and tissue-specific TAAR1-KO or knockdown are needed to obtain clear-cut answers to several crucial questions.

Another methodological limitation affecting many investigations is the absence of a proper comparison between the endogenous concentration of the putative ligand and the EC_50_ derived from pharmacological experiments. In general, the assay of endogenous tissue levels was not adequately validated, and the actual concentrations obtained at receptor level after exogenous administration were not determined.

It must also be realized that the basic biochemical features of this signaling system are still confused or unknown. TAAR1 has the structural features of a plasma membrane receptor, but its physiological location is not completely clear. Most TAAR1 molecules are intracellular, and it is unknown whether this observation reflects subcellular trafficking of membrane receptors, or rather the existence of an intracellular pool of functional receptors, activated by intracellular ligands. The transduction pathway(s) coupled to TAAR1 is (are) also obscure. Gs-mediated cAMP production was initially considered as the hallmark of TAAR1 activation, but we have now evidence that TAAR1 can also activate inward rectifying potassium channels and the β-arrestin 2 pathway, probably by Gs-independent pathways. In addition, a consistent body of evidence support interaction with, and modulation of, other G protein-coupled membrane receptors, possibly mediated by the formation of receptor heterodimers. Putative partners include dopamine, serotonin, adrenergic, and glutamate receptors. In general, the specific pathways activated in specific CNS locations remain to be determined.

If the TAAR1-triggered pathway has a significant modulatory role, one would expect that the concentration and/or availability of its ligands are closely regulated. At present, the brain metabolism of putative TAAR1 ligands is largely obscure. Trace amines are allegedly produced by aromatic amino acids through a few enzymatic steps, always including AADC, but very little is known about the regulation of AADC expression or activity. In the case of T1AM, the matter is more complex, since its synthetic pathway is still controversial, and no evidence of local production within the CNS has been obtained so far (Hoefig et al., 2016).

In conclusion, there is strong circumstantial evidence for an important neurophysiological role of TAAR1, but further investigation is needed to reach definite conclusions. The matter has potential practical implications, since TAAR1 might be an intriguing target for pharmaceutical interventions. In the 16 years elapsed since its discovery, different classes of synthetic TAAR1 agonists have been developed, and promising results have been obtained in experimental models of drug abuse, stress, depression, narcolepsy, and cognitive impairment. Although the history of medicine reports many instances of drugs that were successfully introduced in human therapeutics before their mechanism of action was understood, a deeper knowledge of TAAR1 physiology and pathophysiology would help to focus future pharmacological and clinical research.

## Author Contributions

All the authors contributed to the paper. GR mainly wrote the following sections: TAAR1 expression, cross-talk between TAAR1 and other monoaminergic systems, Transgenic models and human genetic investigations. AA mainly wrote the following sections: receptor pharmacology and Neural effects of TAAR1 ligands. RZ wrote the introduction and conclusions and revised the whole manuscript.

## Conflict of Interest Statement

The authors declare that the research was conducted in the absence of any commercial or financial relationships that could be construed as a potential conflict of interest.
